# Did Ugo Foscolo suffer from chronic renal insufficiency?

**DOI:** 10.1186/s40064-016-1744-1

**Published:** 2016-02-03

**Authors:** Konstantinos Stamatiou, Maria Sgouridou, Georgios Christopoulos

**Affiliations:** Urology Department, Tzaneio General Hospital of Piraeus, Afendouli 1 Ave, 18536 Piraeus, Attica Greece; Department of Italian Language and Literature, School of Philosophy, University of Athens, University Campus, 16121 Athens, Greece; Internal Medicine Department, Tzaneio General Hospital of Piraeus, Afendouli 1 Ave, 18536 Piraeus, Attica Greece

**Keywords:** Ugo Foscolo, Italian literature, Correspondence, Medical history, Renal insufficiency, Cause of death

## Abstract

Ugo Foscolo, was an Italian poet whose works rank among the masterpieces of Italian literature. Talented and well educated in philosophy, classics, and Italian literature, Foscolo gave literary expression to his ideological aspirations and to the numerous amorous experiences in odes, sonnets, plays, poems and an epistolary novel. Concurrent with his rich literary output, Foscolo’s correspondence represents a unique perspective from which to monitor his literary and political views and investigate aspects of his everyday life. Among other interesting information, one can find elements of Foscolo’s medical history which is generally unknown. Based on his testimonies we suggest that he suffered of longstanding bladder outlet obstruction presumably due to urethral stricture. In the present article we investigate the possibility that chronic bladder outlet obstruction and the consequent renal insufficiency was attributed to the death of Ugo Foscolo.

## Background

Ugo Foscolo, was an Italian writer, military, revolutionary, literary and poet whose works are considered among the masterpieces of Italian literature. He was son of Diamantina Spathi who was Greek and Andrea Foscolo who was Venetian. This mixed heritage greatly influenced his life, his political view and his literary production. He spent most of his childhood years in the island of Zakynthos however, when he was 10 years old, his father died and his family moved to Venice. For the time of his residence there he attended political and literary gatherings and soon he developed his own cultural and political profile. Βecause of his political ideas, Foscolo was exiled in Bologna, where he joined the French imperial army against the Austrian invaders (Vincent 1950). In this period (late 1799), he completed his tragedy *Tieste*. In due course he was promoted to captain in the Italian division of the Napoleonic army and was sent to serve in northern Italy and western France (1804–06). During that period he translated some classical works of the ancient Greek literature as well as the *Sentimental Journey* of Laurence Sterne into Italian. He also wrote some odes sonnets, plays and the epistolary novel *The Last Letters of Jacopo Ortis*. In 1807 Foscolo returned to Milan where he composed the poem *On Tombs*. One year later he won the chair of Italian rhetoric at the University of Pavia. As soon as the chair was abolished by Napoleon (1808), Foscolo started composing the tragedies *Aiace* and *Ricciarda*, and the unfinished poem, *Le grazie*. In 1814 Napoleon fell, the Austrians returned to Italy and Foscolo, refusing to take the oath of allegiance, was self-exiled first in Switzerland and then in England. There he wrote commentaries on Dante, Boccaccio, and Petrarch for The Edinburgh Review and The Quarterly Review for livelihood (The Editors of Encyclopædia Britannica 2015). He died of dropsy at Turnham Green near London on Sept. 10, 1827. Few are known about his medical history and cause of death.

Foscolo’s rich correspondence with family, friends, and fellow writers consists of more than 3000 letters. From the ‘Epistolario’ one can monitor his views, study aspects of his everyday life and investigate his medical history which is generally unknown (Parmegiani 2011). In the present article we investigate the nature of the disease that affected Ugo Foscolo and attributed to his death through his correspondence.

## Results

We found several letters reporting on his general health however; there are only eight providing information regarding his medical history and in particular urological diseases. The letter no 1228, to Doctor Luigi Ramondini (Firenze 29 September 1812), the letter no 1230 to Sigismondo Trechi (Firenze, 1 October 1812), the letter no 1236 to Isabella Teotochi Albrizzi (Firenze, 15 October 1812), the letter no 1253 to Francesco Aglietti (November 1812), the letter 1315 to Isabella Teotochi Albrizzi (Bellosguardo 8 June 1813), the letter 1316 to Sigismondo Trechi (Bellosguardo 10 June 1813), the letter 1371 to his mother (Firenze, 23 September 1813) and the letter No. 2291 to his friend John Cam Hobhouse (1 September 1818). According to the information retrieved from the above letters, in September 1812, Foscolo was infected by urethritis. Symptoms gradually disappeared and 8 days later he experienced acute urinary retention which was treated by passing a sound. Dysuria, local pain and mucus overflow occurred since after. Following an episode of gross hematuria the above symptoms were relieved. In the same letter, he reported that he had similar but less painful symptoms 1 year before the onset of “gonorea” (November 1811). This previous infection is also mentioned in the letter nr. 1236. Accordingly to his testimony, the progress of this situation was accompanied by nocturnal bleeding (hematuria). He was practiced dilatation of the urethra using rods of increasing thickness and of variable materials for an unspecified time period. He was also suffering of local pain and fever that were treated with baths and hydrotherapy, bloodletting and Chinese Quinine as well. Foscolo wasn’t able to determine the nature and the precise onset of the disease, however; through his correspondence with Antonietta Fagnani Arese (between 1801 and 1803), became clear that he experienced the above condition many years before. In fact, among almost 200 letters that Foscolo wrote to her there are many mentioning hydrotherapies for pain relief, some reporting febrile flare ups and few presenting Foscolo and Arese to suffer in common from an infectious disease whose nature was not clarified. Correspondence from 1819 to 1826 provides no specific information regarding the above health matter.

According to the information retrieved from the literature the cause of his death is actually unspecified. Most Foscolo’s biographers report that he died on Sept/10/1827 of a long standing liver disease while, Pecchio mention an inflammatory malady of the liver. However among numerous febrile episodes reported by Foscolo there are two characterized as bilious fever (febbre biliosa) [letter no 650 (Aug/12/1826) and letter no 652 (Sept/5/1826)] and one characterized as inflammatory fever (febbre infiammatoria) [letter no 632 (Sept/12/1824)]. Some months before, on 17th April 1824 he reported to Lord Dacre [letter no 627] that he suffered from fever that lasted for several weeks. In the letter no 637 to Mr Walker (Oct. 1824) he mentioned that he had just recovered from a disease. In the letter no 637 to Hudson Gurney (Aug/12/1826) he was seeking of a more effective remedy against the bile, since nor the pills, nor the rhubarb (which he took in high doses) were able to straining the stomach. He also states that he would prefer of any other disease since bilious attacks rendered him oppressively sleepy. In the letter no 652 to Mr Edgardo Taylor (Sept/5/1826), he reported: “when I walked up to my last hovel, I began to suffer of a bilious fever” and “fever turns in what we call cholera morbus. Now I feel better, but I feel very tired, yet, the worst in this disease is a lethargic slumber”. Four months later he writes to Mr Reinaud di Zante [letter no 666 (Jan/9/1827)]: “I was staying for long in bad or mediocre (situation) and never in good health”. Finally, after a short recovery period [letter no 670 (Mar/28/1827)], a febrile (?) disease described as lasting for more than 3 months was reappeared. Accordingly to Antona-Traversi’s notes, Foscolo’s health was getting worst: He wrote to Bossi (Mar/1827) that his fever comes every afternoon and it is associated with bilious irritation. Two months later (Jul/1827) he wrote to Mami that his illness lasts 8 months and was gradually aggravated: Despite frequent bloodletting sessions the fever comes twice daily being slight in the morning and rages during the evening. He also noted that the true nature of this disease was finally revealed as it is a chronic liver and intestinal inflammation. Accordingly to Pecchio’s observations it was not before August 1827 that his health status was irreversibly deteriorated and indeed, towards the end of this month, Foscolo’s disease made life very hard for him. He was losing weight day by day and manifested a predisposition to dropsy as a consequence of a disease of the liver that has long afflicted him. In fact, in the letter no 674, to Canonico Riego (Aug/5/1827), Foscolo notes that the dropsy was growing rapidly however; the surgeon didn’t consider it mature enough for an operation. According to Antona-Traversi, Foscolo finally underwent surgery (paracentesis), which was repeated few days later, however they offered little help. In fact, Foscolo wrote to Bossi (Aug/21/1827) that weakness, insomnia and dyspepsia continued while dropsy relapsed as well. In Sep/7/1827 he went into a coma and died on Sep/10/1827.

## Discussion

The term dropsy (also hydropsy) describes the edematous accumulation of fluid in the interstitium. The amount of interstitial fluid is determined by the balance of fluid homeostasis. When the last is disrupted—either by the increased secretion or the impaired removal of the fluid into and from the interstitium—can cause edema. The typical clinical manifestation is whole-body swelling. Situations that alter the balance of fluids are cardiac, kidney and liver failure (Vinay et al. 2006). None of the above is clearly mentioned in the correspondence. In contrast, most authors report that he died of a long standing liver disease whose nature and beginning are not clear.

Actually, in the eighteenth century, the term dropsy was referred to preternatural swelling of the whole body, or some part of it, occasioned by a collection of watery humour. It was distinguished by different names, according to the part affected, as the anasarca, or a collection of water under the skin; the ascites, or a collection of water in the belly; the hydrops pectoris, or dropsy of the breast etc. (Buchan 1785). It was not until 1871 that dropsy was considered as a sequel of many chronic diseases, particularly those of the kidneys (Murchison 1871). Between 1820 and 1830, Richard Bright, an English physician and early pioneer in the study of kidney pathophysiology, extensively researched kidney disease, along with diseases of the heart, liver, pancreas and pulmonary system. He demonstrated a variety of kidney disorders having high concentrations of protein in the urine (Kinder 1966). Until that time, many people suffering from kidney diseases went undiagnosed although it was recognized then that they died of urea poisoning, or “dropsy”.

Whether and if Foscolo suffered of a kidney disease remains unknown since no available information exists. Evidence from his correspondence suggests that he may gradually developed chronic renal insufficiency due to chronic urinary obstruction. In fact, in September 1812, Foscolo reports that he was infected by a sexually transmitted disease (STD). Of note, the term gonorea (gonorrhoeae) used by him merely describes the usual symptoms of STDs such as burning with urination and penile discharge than refers to infection by Neisseria gonorrhoeae (unknown at that time since it was first discovered in 1879). Ιnfection was followed by inflammation and urinary retention. Gradually pain and lower urinary tract symptoms (both storage and voiding) were established. The exact disease from which Foscolo suffered is not mentioned in his correspondence however many common diseases and conditions were practically unknown from medicine of that time.

Today, it is well known that an important number of patients with gonococcal urethritis develop urethral stricture. Especially those with posterior strictures are in higher risk to develop acute urinary retention (Mundy 2006). In fact, without appropriate treatment, the infection extends beyond the urethral mucosa to the corpus spongiosum causing surrounding tissue necrosis and granulation tissue formation. Subsequently, fibrous scarring develops in the affected area which progressively increases the resistance to micturition causing thus bladder instability. Several patients develop epididymitis and prostatitis as a result to the expansion of the infection to the surrounding structures. Both stricture and prostatitis cause frequency of micturition, pain, urinary retention and hematuria. Actually, these are exactly the symptoms that Foscolo describes in the letter nr. 1230. At that time, intermittent self-dilatation was considered a convenient way to treat urethral strictures and in fact, between 1813 and 1818 he used cylindrical urethral dilatators in order to treat bladder outlet obstruction. However, it is now generally accepted that dilatation is moderately effective and can be expected to cure about 50 % of short bulbar urethral strictures when first used. If the procedure has to be repeated, it is rarely curative and it is rarely curative even the first time in strictures other than in the bulbar urethra (Devereux and Burfield 1970). When the stricture recurs, it usually does so within weeks or months and almost always within 2 years. Those that recur comparatively infrequently might be palliated (rather than cured) by repeat instrumentation (Devereux and Burfield 1970).

The fact that, between 1813 and 1818, Foscolo’s correspondence includes information regarding urethral strictures treatment indicates a longstanding bladder outlet obstruction. In fact, if instrumentation is required more frequently or is complicated, then chronic retention and inflammation are established. The expanding inflammation may result in inflammatory-mediated pain and fever and is usually characterized by flare-ups and remissions (Mundy 2006). Whether or not this situation progressed to chronic obstructive uropathy remains unknown since correspondence from 1819 to 1826 provides no specific information regarding the above health matter. Ιf chronic obstructive uropathy occurred, associated symptoms such as febrile episodes, vomiting and edema can be attributed to urinary tract infection, uremia and hypoproteinemia respectively.

Of note, numerous febrile episodes are mentioned in the correspondence of Foscolo since the presumed onset of the disease. The term bilious fever (febbre biliosa) used by him is not necessarily associated with a disease of the liver. Actually, “bilious” means the condition was thought to arise from disorders of bile, the two types of which were two of the four humours of traditional Galenic medicine in 200 A.D., and bilious fever was a medical diagnosis often used for any fever that exhibited the symptom of nausea or vomiting in addition to an increase in internal body temperature. The term is no longer used, but was used by medical practitioners in the eighteenth and nineteenth centuries. Among various definitions for the term bilious fever that have emerged over the past centuries is that of Buchan (1785): “continual, remitting, or intermitting fever accompanied with a frequent or copious evacuation of bile, either by vomit or stool”, of Dunglison (1855): “common remittent fever of summer and autumn” or that of Appleton (1904): “Typhoid fever, remittent fever or simple gastritis”. Similarly, the term cholera morbus used by Foscolo is not obliquely associated with an infection of the small intestine caused by the bacterium Vibrio cholera, since at that time it was used to describe acute gastroenteritis occurring in summer and autumn marked by severe cramps, diarrhea, and vomiting (Wilson 1832).

Modern diagnoses for the same symptoms would include a wide range of conditions and infections including obstructive uropathy. Interestingly, consequences of urine retention were known at that time: “When the urine is too long retained, it is not only resorbed, or taken up again into the mass of fluids, but by stagnating in the bladder it becomes thicker, the more watery parts flying off first, and the more gross and earthly remaining behind. By the constant tendency which these have to concrete, the formation of stones and gravel in the bladder is promoted […] Many persons have lost their lives, and others have brought on very tedious, and even incurable disorders, by retaining their urine too long, from a false delicacy. When the bladder has been over-distended, it often loses its power of action altogether, or becomes paralytic, by which means it is rendered unable either to retain the urine, or expel it properly. The calls of Nature ought never to be postponed. Delicacy is doubtless a virtue; but that can never be reckoned true delicacy, which induces any one to risk his health, or hazard his life” (Buchan 1785).

It therefore remains unclear how Foscolo, his doctors and his biographers missed this point. Given the long-stand- ness of the urethral structure treatment it is less likely that obstruction was spontaneously resolved. The fact that references to urological problems do not appear in Foscolo’s correspondence from 1819 onward could be associated to an improvement of his lower urinary tract symptoms. It should be noticed that the management of urinary tract infections at that time included hospitalization, bed rest, attention to diet, plasters, narcotics, herbal enemas and douches and judicious bleeding (direct bleeding, cupping and leeches). Most of the above mentioned treatments are practically ineffective. Foscolo was treated with baths and hydrotherapy, bloodletting and Chinese Quinine as well. Quinine is an alkaloid derived from the bark of the cinchona tree. It has been used as an antimalarial drug since before 1633. It has also antipyretic, analgesic, and anti-inflammatory properties and at that time it has been used in common cold preparations for that purpose. It reduce the sensitivity of muscle cells to stimuli that cause them to contract, and prolong the time it takes for the muscle to contract. The above properties of Quinine suggest effectiveness in the treatment of prostatitis related pain and storage lower urinary tract symptoms, however reduced contraction rate may progress to chronic urinary retention (especially when bladder outlet obstruction occurs). Moreover, Foscolo used to eat excessive quantities of rhubarb and drink its extract in order to treat his illness. The leaves of this vegetable contain high levels of oxalic acid, which causes serious problems in the liver and kidney and even a small amount is sufficient for someone to get sick.

## Conclusions

In conclusion, the medical history of Ugo Foscolo is poorly investigated. While most authors report that he died of a long standing liver disease, its nature and beginning are not clear. Evidence from his correspondence suggests that he suffered of chronic bladder outlet obstruction and he may gradually developed chronic renal insufficiency that probably attributed to his death.

## Methods

We conducted a research on Ugo Foscolo’s correspondence -included in the Epistolario- in order to identify information regarding his medical history focusing mainly on urological diseases and relative conditions. Initial search terms were “*vescica*”, “*prostata*”, “*uretra*”, “*medico*”, “*chirurgo*” combined with “*malatia*”, “*infermita*”, “*irritazione*”, “*terapia,* “*salute*”, “*infiammazione*”. The text was studied in original language (Italian or French), from: Ugo Foscolo, Epistolario, Edizione Nazionale delle Opere di Ugo Foscolo, Le Monnier, Firenze 1954.

Information regarding his death were retrieved from his correspondence (Epistolario) and the journals “The Gentleman’s Magazine”, (A. Dodd and A. Smith eds., 1827; vol. XCVII, part 2; page 566), “The New Monthly Magazine and Literary Journal” (H. Colburn ed., 1832; vol. XXXIV., no. CXXXIII, page 168), “The Museum of Foreign Literature, Science and Art” (E. Littell ed., 1827; vol. XI, pp. 557–560), and the books “Divagazioni letterarie” (G. Surra, tip. Guaglio, Novara, 1911, pp. 76–105), “Ugo Foscolo: An Italian in Regency England” (E. R. Vincent, Cambridge: At the University Press, 1953, p. 210) and “Vita di Ugo Foscolo” (G. Pecchio, ed. D. G. Rossi, Genova 1853 pp. 167–171). Information related to dropsy was investigated in medical textbooks of that time such as: “Bilious Inflammatory Fever” (G. Perkinson, Printed By Woody W., Baltimore, 1822), “The Causes, Treatment, and Cure of Fever and Ague, and Other Diseases of Bilious Climates” (C. Osgood, New York, 1865), “Collection Of Papers On The Subject Of Bilious Fever” (Webster N., Printed By Hopkins, Webb And Co. New York, I796), “A Text-Book with Particular Reference to Physiology and Pathological Anatomy” (F. Von Niemeyer, H. Humphreys G and C.E. Hackley ed, New York, 1870).
